# Failure Behavior of Nano-Metakaolin Concrete Under Splitting Tension Based on Digital Image Correlation Method

**DOI:** 10.3390/polym16243482

**Published:** 2024-12-13

**Authors:** Hao Chen, Yingfang Fan, Qiuchao Li, Chang Peng

**Affiliations:** Institute of Road and Bridge Engineering, Dalian Maritime University, Dalian 116026, China; ch0120230106@dlmu.edu.cn (H.C.); lqc9508@163.com (Q.L.); p1597795@163.com (C.P.)

**Keywords:** nano-metakaolin, failure behavior, digital image correlation, interface transition zone

## Abstract

Nano metakaolin (NMK) has attracted considerable interest for its potential to improve the durability of cementitious materials. However, the effect of NMK on the splitting tensile performance of concrete has not been systematically investigated. This study investigates the splitting tensile performance of NMK concrete and analyzes its failure behavior under splitting load. Different NMK dosages (0%, 1%, 3%, 5%, and 7%) were considered, and splitting tensile tests were conducted. The crack propagation process, crack width, and crack growth rate on the surface of NMK concrete during the splitting tensile test are analyzed using the Digital Image Correlation (DIC) method. The mechanisms by which NMK affects the splitting tensile performance of concrete were examined using X-ray Diffraction (XRD), Fourier Transform Infrared Spectroscopy (FT-IR), Scanning Electron Microscopy/Energy Dispersive Spectroscopy (SEM/EDS), and Thermogravimetric Analysis (TG). The results indicate that the incorporation of NMK enhances the splitting tensile performance of concrete. Concrete with 5% NMK addition exhibited the highest splitting tensile strength, with an increase of 17.4% compared to ordinary concrete. NMK improved the cracking resistance and overall integrity under splitting tensile load. With 5% NMK addition, the surface crack length, width, and main crack propagation rate of the concrete decreased by 4.5%, 35.3%, and 29.6%, respectively. NMK contributed to a denser internal structure of the concrete, promoted the formation of C-S-H gel, and increased the degree of cement hydration. Moreover, a lower thickness and Ca/Si ratio of interfacial transition zone (ITZ) were observed in NMK concrete. The ITZ thickness and Ca/Si ratio of concrete with 5% NMK were reduced by 64.4% and 85.4%, respectively, compared to ordinary concrete. In summary, the influence mechanism of NMK addition on the splitting tensile strength and failure behavior of concrete is explored in this study, providing experimental data to support the application of NMK concrete in practical engineering.

## 1. Introduction

Concrete is extensively used in construction, with its mechanical properties being critical to the safety and durability of structures. However, large amounts of microscopic cracks and pores form within the concrete, becoming initiation points for crack propagation under tensile loading, resulting in weak crack resistance [1,2]. Furthermore, the weak bond between the mortar and aggregates reduces the capacity of concrete to resist stress concentration and load transfer, contributing to its relatively low tensile strength compared to compressive strength [3]. Hence, enhancing the tensile properties of concrete is critical for ensuring structural safety and performance under diverse load conditions.

In recent years, nano materials such as carbon nanotubes (CNT) [4,5], graphene (GO) [6], and nano silica (NS) [7,8,9] received more attention due to their pozzolanic effect, pore filling and nucleation effect [10,11,12,13,14]. Previous researchers have investigated the effect of various nanomaterials on the tensile performance of concrete. The results reveal that nanomaterials addition significantly enhance the tensile properties of concrete. Liu et al. [4] discovered that the ultimate tensile strength of concrete increased by 8.5% with the incorporation of 0.025 wt.% CNT compared to conventional concrete. Song et al. [5] found that adding 0.2 wt.% CNT could enhance the tensile strength at the aggregate-mortar interface by 8.4%. Wu et al. [6] reported that the inclusion of 0.03% GO in concrete resulted in a 24.8% improvement in splitting tensile strength compared to ordinary concrete. Amin et al. [7] observed that the addition of NS led to a 44% increase in the splitting tensile strength of concrete compared to conventional concrete. Mazloom et al. [8] and Ashokanet al. [9] similarly observed that the incorporation of NS enhances the tensile properties of concrete. Although nanomaterial concrete exhibits superior tensile strength, it is also associated with challenges, including high production costs [15] and potential reductions in long-term durability. Compared to above nanomaterials, NMK shows substantial promise as a supplementary cementitious material, especially due to its lower cost and its effectiveness in promoting the durability [16] of cement-based materials [13,14,17,18]. Previous studies showed that, compared with ordinary cement mortar, the crack resistance of cement mortar mixed with 5% NMK was significantly improved, and the cracking load increased by 11.4% and 11.0% at 3 d and 7 d [13], respectively, and the fracture energy increased by three times at 28 d [18]. The incorporation of 5% NMK into the concrete resulted in a notable enhancement in compressive strength, with a 38.0% increase observed at 3 days and a 17.0% improvement at 28 days, in comparison to conventional concrete [19]. In addition, Xie et al. [17] enhanced the recycled aggregate concrete mechanical performance by using NMK. The result found that the reclaimed concrete showed the maximum compressive strength of 5% NMK, which increased by 17.0% compared with ordinary reclaimed concrete. The findings suggest that NMK is an ideal supplementary cementitious material, due to its positive effects on enhancing both the durability and mechanical properties of concrete. Although extensive research has been conducted on the mechanical properties of NMK concrete, research on its tensile performance remains limited. Thus, a comprehensive study of the tensile performance and failure behavior of NMK concrete is essential, both from a theoretical perspective and for its practical implications.

Concrete, as a non-uniform composite material, exhibits complex cracking behavior that is particularly evident under loading and failure. Therefore, understanding its failure behavior is crucial for improving the mechanical properties of concrete [20,21]. Traditional measurement methods for analyzing concrete failure behavior often require prior knowledge of crack locations and propagation paths. However, concrete’s failure behavior usually manifests as micron-scale cracks randomly distributed on its surface, posing significant challenges for analysis using traditional contact-based measurement techniques. Digital Image Correlation (DIC) is a non-destructive testing method [22,23,24,25] that can continuously capture images of the deformation process on the concrete surface. By processing and analyzing these images, the deformation and strain distribution on the surface under load can be obtained.

Sutton et al. [23] optimized the Digital Image Correlation (DIC) method by minimizing the search time and combining the Newton–Raphson method with the differential correction technique. The optimized approach was found to be 20 times faster and more accurate in displacement measurements compared to the conventional DIC methods. Due to the advancements in DIC technology [26,27], it has become widely used for monitoring the surface deformations of concrete under applied loads.

Li et al. [13] analyzed the early cracking process of NMK cement mortar using DIC method and evaluated its early fracture behavior. Yang et al. [2] measured the surface horizontal strain of basalt fiber-reinforced concrete (BFRC) and analyzed its splitting failure characteristics. Xie et al. [28] determined the crack propagation length during the fracture process of geopolymer concrete using DIC method. Suchorzewski et al. [29] used DIC to investigate the crack evolution in CNT concrete during splitting tensile tests. DIC has significant advantages in analyzing concrete failure behavior, overcoming the limitations of traditional methods, and more accurately capturing and characterizing the development and propagation of micro-cracks in concrete under load [28,29].

In summary, most studies focus on the effects of NMK on the mechanical properties of concrete, with limited attention given to the failure behavior during the testing process and the enhancement mechanisms of NMK on the tensile strength of concrete. Thus, in this study, the effects of five different dosages of NMK on the splitting tensile strength of concrete was examined initially through splitting tensile tests. The failure behavior during the splitting testing of NMK concrete was characterized using the DIC method. Finally, the mechanisms by which NMK enhances the splitting tensile strength of concrete were analyzed using microscopic techniques such as SEM, EDS, and TG, providing valuable guidance for the further application of NMK in enhancing concrete performance.

## 2. Materials and Methods

### 2.1. Materials

**Figure 1:**
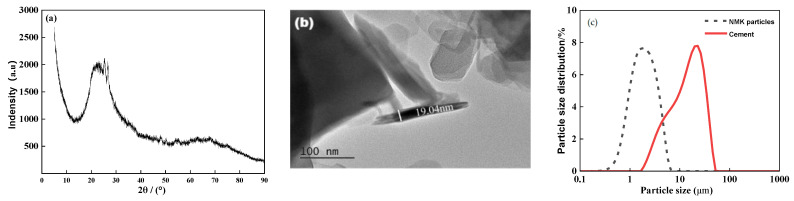
The physical index of materials. (**a**) XRD pattern; (**b**) TEM morphology; (**c**) particle size distribution.

In this study, ordinary Portland cement (P·O 42.5R), produced by Dalian Cement Group Co., Ltd. (Dalian, China), was selected. Nano-metakaolin (NMK) was utilized as an admixture in the concrete, replacing cement by mass at dosages of 0%, 1%, 3%, 5%, and 7%. The physical properties of NMK are listed in Table 1, and its XRD pattern and TEM morphology are shown in Figure 1a,b, respectively [13,30]. The chemical compositions of the cement and NMK are presented in Table 2. Crushed stone with a particle size range of 5–25 mm was utilized as the coarse aggregate. Medium-coarse river sand with a fineness modulus of 2.58 was used as the fine aggregate, with a bulk density of 1347.54 kg/m^3^ and a mud content of 4.4%. Tap water was used as the test water. The particle size distributions of cement, sand, and NMK are shown in Figure 1c. The mix proportions used for preparing NMK concrete are listed in Table 3.

### 2.2. Specimens Preparation

**Figure 2:**
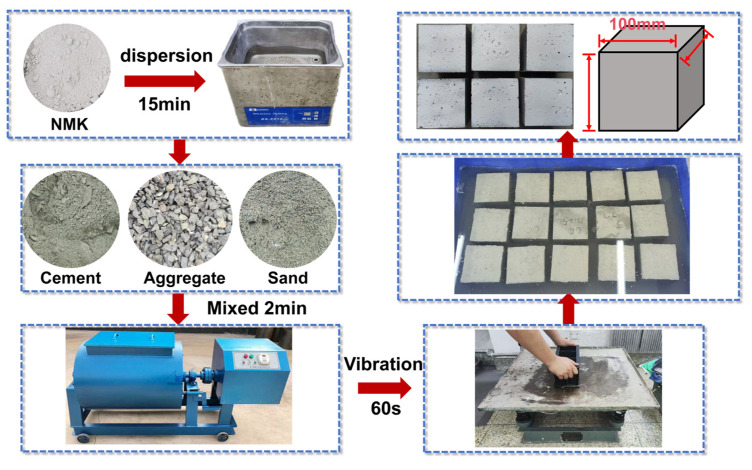
Preparation of NMK concrete.

To investigate the effect of NMK on the mechanical properties of concrete, a total of 30 concrete cube specimens with dimensions of 100 mm × 100 mm × 100 mm were cast, as illustrated in Figure 2. The process of NMK concrete preparation are as follows. Initially, cement, sand, and coarse aggregate were preliminarily mixed for 60 s through a concrete mixer. NMK and water were poured into an ultrasonic cleaner for dispersion, with the duration of the process being 15 min [13], to ensure uniform distribution of NMK in the water and to prevent agglomeration. Following the preparation of the NMK suspension, it was introduced into the concrete mixer and mixed for an additional 60 s. Once the mixing procedure was completed, the fresh NMK concrete were cast into steel molds in two layers. Subsequently, a thin layer of plastic film was applied to cover all samples, thereby preventing the evaporation of moisture. After 24 h, the samples were demolded from the steel molds. Finally, the specimens were placed into water (temperature = 20 ± 2 °C; relative humidity = 98% RH) for continued curing 28 d prior to mechanical testing.

### 2.3. Experiment Method

#### 2.3.1. Splitting Tensile Test

**Figure 3:**
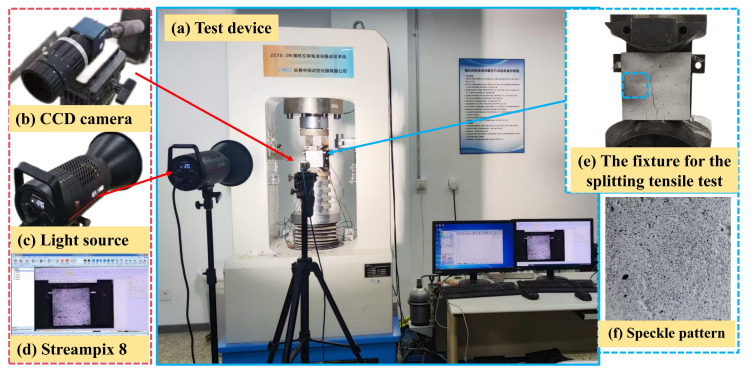
The setup of the compressive and splitting tensile strength tests.

The splitting tensile strength test for concrete was conducted in accordance with the current standard GB/T 50081–2019 [31], using an electro-hydraulic servo testing machine. Cubic concrete specimens with dimensions of 100 mm × 100 mm × 100 mm were tested after a curing period of 28 d. The ZCTS-2M(Zhongce Testing Instrument Corporation, Changchun, China) electro-hydraulic servo press, with a maximum loading capacity of 2000 kN, was utilized for testing, as illustrated in Figure 3a. The device system for the splitting tensile test is illustrated in Figure 3a–e. The test setup involved placing the specimen horizontally between the loading platens of the machine. A strip of plywood or other suitable packing material was placed between the platen and the specimen to ensure uniform load distribution and to avoid stress concentrations, as shown in Figure 3e. The splitting tensile strength tests were performed at a loading rate of 0.1 mm/min [2]. Three specimens were tested for each group, and the splitting tensile strength of each specimen was recorded. The average splitting tensile strength of the three specimens was taken as the final splitting tensile strength of that group of concrete. The splitting tensile strength was calculated according to Equation (1) [31]:

(1) $$f_{\mathrm{st}}=\frac{\alpha \times 2F_{\mathrm{st}}}{\pi A},$$

where

*f*_st_ = the splitting tensile strength of NMK concrete (MPa);
*F*_st_ = the maximum splitting tensile load applied to the specimen (kN);
*A* = the loaded area of the concrete specimen (mm^2^);
*α* = Size effect conversion coefficient, which value is 0.85.

#### 2.3.2. Digital Image Correlation Method (DIC)

DIC method quantitatively analyzes the deformation, motion, or structural changes in objects by comparing feature points across two or more sequential images. The core mechanisms of DIC include feature extraction, feature matching, and displacement evaluation, which involved algorithms calculating the relative positional changes in feature points between different states to derive the displacement, deformation, or motion patterns of the object [22,23]. This non-contact measurement method is extensively utilized in the analysis of the mechanical properties of concrete. DIC offered a highly efficient and precise analytical tool for evaluating surface deformation, crack formation, and propagation in concrete, as well as for assessing structural deformation and damage.

Therefore, a DIC system was employed to investigate the failure behavior of NMK concrete, as shown in Figure 3b–d. The DIC testing system consists of three components: a high-speed CCD camera, a light source (Figure 3d), and a data acquisition system. The MER-230 camera (Daheng Corporation, Beijing, China) is used to capture images of the concrete surface during the splitting tensile test, with a resolution of 1920 × 1200 and a maximum acquisition frequency of 168 fps, as shown in Figure 3b. The acquisition process is controlled by the Streampix 8 system, developed by Norpix Inc. (Montreal, QC, Canada), as illustrated in Figure 3d. Due to the non-uniform color distribution on the concrete surface, directly applying black speckles is not conducive to the accurate acquisition of surface deformations using the Digital Image Correlation (DIC) method [13,28]. Therefore, to highlight the speckle pattern on the concrete surface and ensure the accuracy of the DIC method, a layer of white paint is initially applied to the surface. Once the white paint has dried, black speckles are randomly added, as shown in Figure 3f. The working distance of the camera was set to 80 mm, ensuring that the specimen was fully within the camera’s field of view. During the splitting tensile test, the capture rate was 10 fps. The surface images of the specimens were processed using DIC-3D 2020 software to obtain information on surface deformation and crack propagation.

#### 2.3.3. Micro-Structure Test

**Figure 4:**
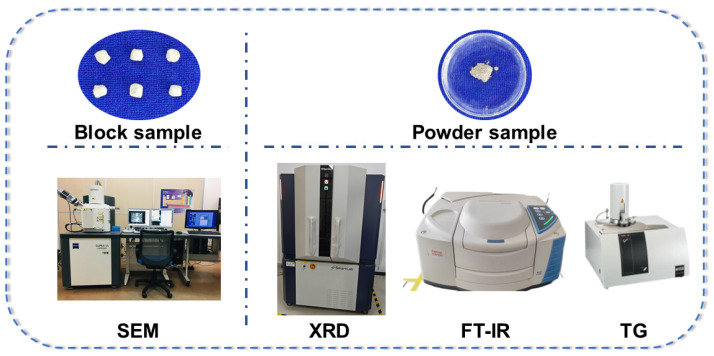
The test devices and the required samples.

Scanning Electron Microscopy (SEM), Energy Dispersive Spectroscopy (EDS), X-ray Diffraction (XRD), Fourier Transform Infrared Spectroscopy (FT-IR), and Thermogravimetric Analysis (TG) were employed to investigate the micro-enhancement mechanisms of NMK in concrete. The test devices and the required samples are shown in Figure 4.

Block samples with dimensions of approximately 10 mm × 10 mm × 10 mm were prepared for SEM testing, which were extracted from the central part of crushed concrete specimens. A field-emission scanning electron microscope (SEM) Tescan Mira3 (Tescan Corporation, Brno, Czechia) was utilized to analyze the microstructure of the interfacial transition zone (ITZ) between the aggregate and mortar in the specimens. To terminate the cement hydration process, the specimens were immersed in anhydrous ethanol for a period of 24 h and subsequently subjected to drying at a controlled temperature of 40 °C in a desiccator until they reached a constant mass. To enhance the electrical conductivity of the specimens prior to SEM analysis, a gold-coating process was employed to deposit a thin layer of gold onto the surface of the specimens.

Powdered samples were prepared for XRD, FT-IR, and TG tests. Several fragments from the center of crushed concrete specimens were placed in a mortar and ground into powder. Anhydrous ethanol was continuously added during the grinding process to halt hydration. The samples were then placed in an oven and dried at a constant temperature of 40 °C until a constant weight was achieved, followed by sieving through a standard sieve with a mesh size of 0.065 mm. The crystalline phases and compositions of the NMK concrete samples were analyzed using XRD, employing a Japanese Rigaku D/MAX-2600 X-ray diffractometer (Rigaku Corporation, Tokyo, Japan). Copper Kα radiation (λ = 1.5418 Å) was utilized for the XRD tests, with a scanning angle range of 5° to 90° and a scanning speed of 5°/min. The functional groups and chemical bonds in the NMK concrete samples were analyzed using FT-IR spectroscopy with a Thermo Fisher Scientific Nicolet iS20 instrument (Thermo Fisher Scientific Corporation, Waltham, MA, USA). The wavelength range for the FT-IR experiments was set from 400 cm^−1^ to 4000 cm^−1^. The content of calcium hydroxide and C-S-H gel in the NMK concrete samples was analyzed using TG with a Netzsch thermogravimetric analyzer (Netzsch Corporation, Bavaria, Germany) from Germany. During the test, the samples were heated from ambient temperature (30 °C) to 900 °C at a heating rate of 10 °C/min.

## 3. Results and Discussion

### 3.1. Splitting Tensile Strength

**Figure 5:**
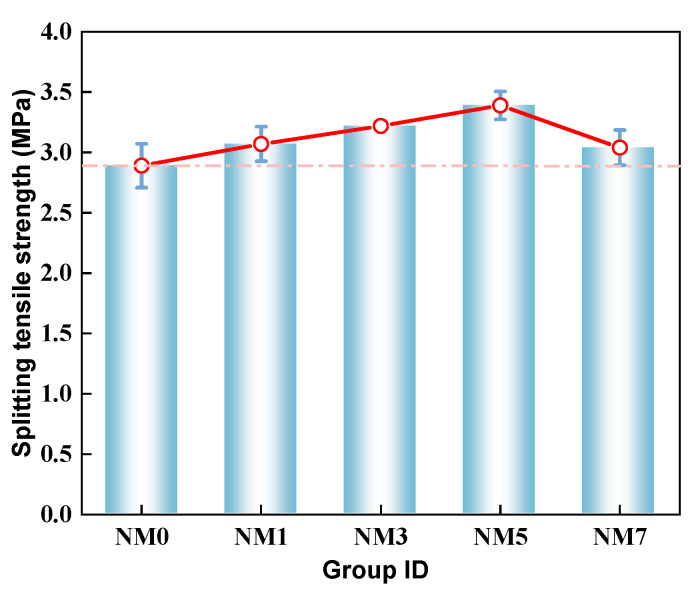
The splitting tensile strength of NMK concrete.

The splitting tensile strength of NMK concrete is depicted in Figure 5. The results demonstrate that NMK addition significantly increases the splitting tensile strength of concrete. Among all specimens, NM0 exhibited the lowest splitting tensile strength of 2.89 MPa. With the increase in NMK content, the splitting tensile strength of NMK concrete exhibited an initial increase followed by a decline. Significantly, the NM5 specimen achieved the highest splitting tensile strength, representing a 17.4% enhancement compared to NM0. The improvement is mainly due to the high pozzolanic activity of NMK [13,17], which promotes the secondary hydration process in cement, leading to the formation of more hydration products (C-S-H and C-A-S-H), thus improving the splitting tensile strength of the concrete. Furthermore, the formation of additional C-S-H gel and C-A-S-H gel acted as bridges between the mortar and aggregates and effectively transmitted tensile stress [32,33], thereby inhibiting the generation and propagation of microcracks [13,18]. Nevertheless, when the NMK content surpassed 5%, the splitting tensile strength of the NM7 showed a 10.2% reduction compared to NM5. This reduction in splitting tensile strength is attributed to the agglomeration of NMK particles [17], increasing concrete porosity and further intensifying stress concentration within the concrete under load.

### 3.2. Failure Behavior of NMK Concrete

#### 3.2.1. Splitting Tensile Load-Displacement Curves

**Figure 6:**
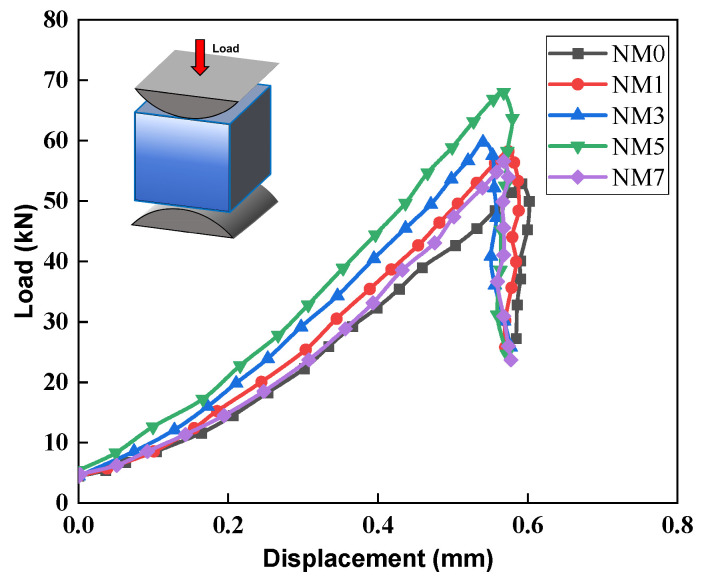
Splitting tensile load-displacement curves of NMK concrete.

The load-displacement curve for NMK concrete under splitting tensile load is presented in Figure 6. The result reveals that NMK concrete demonstrates pronounced brittle failure characteristics, as the load rapidly declines to the minimum value after reaching the ultimate load, leading to splitting tensile failure. The peak displacement of NMK concrete primarily remains approximately 0.6 mm, showing no significant change with increasing NMK content. In contrast, a significant increase in ultimate load is observed as NMK content increasing. This suggests that NMK does not improve the ductility of concrete. Moreover, a steeper ascending slope was observed in NMK concrete compared to the NM0. The ability of NMK concrete to resist deformation increases with the slope of the load-displacement curve in the linear stage. Thus, the slope of the NMK concrete curve within the 0–0.2 mm range is calculated, as the curve within this range exhibits a nearly linear increase. Specifically, the slopes for NM1, NM3, NM5, and NM7 are 56.6 kN/mm, 69.4 kN/mm, 73.8 kN/mm and 55.6 kN/mm, indicating increases of 15.7%, 41.9%, 50.9%, and 13.7%, respectively, compared to NM0. It is evident that NMK concrete shows smaller deformations during the stage of increasing splitting load. This indicates that the incorporation of NMK significantly improves the integrity of concrete and inhibits the development of cracks within concrete. However, excessive NMK addition results in a clear reduction in the slope of the concrete, which could be a potential reason for the decrease in splitting tensile strength observed in the NM7.

#### 3.2.2. Failure Behavior of NMK Concrete

##### Surface Crack Propagation in NMK Concrete

**Figure 7:**
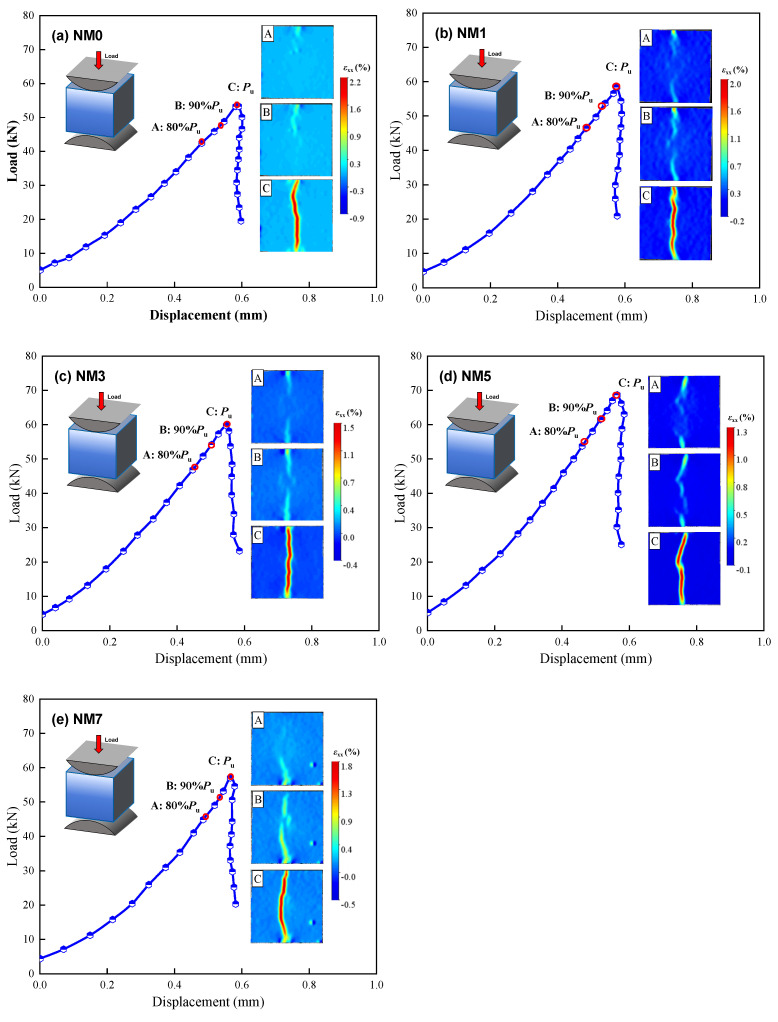
The strain clouds of NMK concrete surface at different loading stages.

To further investigate the effect of NMK on the splitting tensile failure behavior of concrete, crack patterns under varying stress ratios were captured using the DIC method. The stress ratio refers to the ratio between the current stress and the ultimate stress. No noticeable changes in surface strain were found when the stress ratio was below 0.8, which consistent with previously reported findings. Hence, the transverse strain (ε_xx_) distribution at stress ratios of 0.8, 0.9, and 1.0 was selected for analysis. Figure 7 illustrates the surface strain evolution in concrete with different NMK contents. Red indicates areas with high tensile strain, typically associated with larger cracks, while blue represents regions under compressive strain. The transition from blue to red reflects the progression of crack development. NMK concrete shows a similar crack propagation pattern. Prior to reaching 80% of the ultimate load (P_max_), the surface strain distribution is relatively uniform, with low value of strain. As the load increases, distinct strain concentration zones emerge at the top and bottom of the specimen, indicating the beginning of microcrack formation and expansion [34]. Upon reaching 90% of P_max_, these concentrated strain areas extend, following a winding path. This tortuous path is a result of stress concentration in the aggregate-mortar interfacial transition zone (ITZ), leading to new crack formation. These microcracks further expand and propagate through the concentrated strain zones, eventually resulting in NMK concrete failure [34,35]. The ITZ exhibits weak crack resistance, which, under stress concentration, leads to the easy formation of new cracks within the ITZ. This leads to cracks mainly propagating along the aggregate edges, resulting in a tortuous path. These microcracks then expand and permeate through strain concentration zones, ultimately causing a tensile failure of NMK concrete.

Comparing the crack propagation of NMK concretes shows that NMK content has a distinct impact on the crack paths on the concrete surface during splitting tensile testing. With increasing NMK content, the crack paths become more linear. This is because NMK improves the crack resistance of the ITZ, enhancing the tensile strength of the concrete. Specifically, NMK enhances the bond between the mortar and aggregates, preventing stress concentrations from deflecting cracks along the ITZ [14,17]. Consequently, cracks are more likely to pass directly through the aggregates, resulting in straighter paths [2,34]. Furthermore, the incorporation of NMK reduced the maximum *ε*_xx_ value in the strain concentration region when it penetrated through the surface of the concrete specimen. When the strain concentration region penetrated into NM5, the maximum surface strain reached 1.32%, representing a reduction of 39.4%, 34.7%, 9.6%, and 26.3% compared to NM0, NM1, NM3, and NM7, respectively. This indicates that the addition of NMK enhances the deformation capacity of concrete under load, enhancing the overall integrity of NMK concrete [34].

##### Rate of Crack Propagation

**Figure 8:**
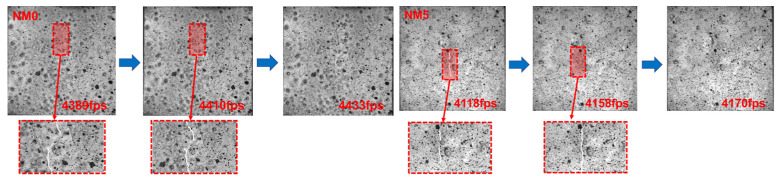
The process of initiation, propagation, and penetration of main crack.

A crack that traverses the surface of the specimen, with its propagation predominantly along the vertical direction, is commonly identified as the main crack. This crack is a key factor in the occurrence of splitting failure in concrete. Therefore, analyzing the initiation and propagation processes of the main crack is beneficial for further investigation into the failure behavior of NMK concrete [36,37]. As illustrated with NM0 and NM5, the processes of main crack initiation, expansion, and penetration are depicted in Figure 8. Under the splitting load, the main crack in NM0 was initiated near the upper end of the specimen. As the load increased, the crack continued to extend and eventually traversed the cross-sectional area of the specimen. As the load increased, the main crack gradually propagated, with its propagation path primarily following the height direction of the specimen, ultimately penetrating through to the bottom of the specimen. The duration of crack propagation processed of NM0 was 44 fps. NM5 demonstrated a longer duration for crack propagation (52 fps), increasing by 18.2% compared to NM0. However, despite the longer duration of crack propagation in NM5, the brittle failure characteristics of concrete under the splitting load were still evident.

The above analysis has demonstrated that NMK significantly affects the propagation process of the main cracks in concrete. Consequently, the main crack propagation in concrete with varying NMK contents was compared and analyzed using the DIC-3D software and the Matlab R2024a crack length identification toolbox. The main crack lengths and their relative propagation velocities in NMK concrete, as calculated, are summarized in Table 4. With the increase in NMK content, the length of the main cracks in concrete gradually decreases. Compared to NM0, the main crack lengths in NM1, NM3, NM5, and NM7 were reduced by 2.8%, 2.7%, 4.5%, and 5.2%, respectively. These findings are consistent with the results observed in the DIC analysis. Furthermore, as the dosage of NMK increases, the propagation rate of the main cracks initially decreases and then increases. NM5 exhibits the lowest rate of crack propagation, reducing it by 29.6% compared to NM0. This is attributed to the formation of a dense 3D network structure by NMK with hydration products such as C-S-H and C-A-S-H [13,17], which is embedded in the ITZ, increasing the energy required for crack propagation and thus enhancing the crack resistance of NMK concrete and reducing the rate of crack propagation. Nevertheless, the rate of crack propagation of NM7 is essentially consistent with that of NM0. This is mainly attributed to the aggregation of NMK, which increases the internal porosity of the concrete. This higher porosity makes the specimen more susceptible to stress concentration during crack propagation, resulting in the formation of microcracks and a decrease in the crack resistance capabilities of concrete.

#### 3.2.3. Distribution Characteristics of Splitting Cracks Based on Fractal Dimension

**Figure 9:**
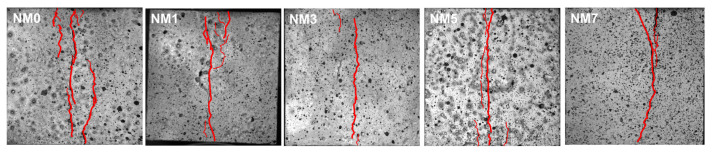
Failure crack pattern of NMK concrete.

Figure 9 illustrates the typical characteristics of splitting failure in concrete with different dosages of NMK. The distribution of surface cracks in NMK concrete exhibits significant randomness, which is associated with the inhomogeneity of the concrete composition. The splitting cracks are predominantly distributed around the central line of the specimen, with the most extensive crack identified as the main crack, which is the principal reason for the splitting failure of concrete. Characteristically, the main crack extends through the surface of specimen in the length direction and primarily propagates parallel to the loading direction. The crack widths in NMK concrete were obtained through a crack microscope. NM5 exhibited the smallest main crack width of 0.224 mm, a 35.3% reduction compared to NM0 (0.346 mm). This finding indicates that NMK plays a role in inhibiting crack propagation, subsequently improving the overall performance of the concrete [35]. Additionally, a few short and narrow secondary cracks were observed around the main cracks, which are microcracks resulting from stress concentrations in the weakened areas around the cracks. As the dosage of NMK increases, a noticeable reduction in the number of cracks on the specimen’s surface is observed. This suggests that the incorporation of NMK enhances the crack resistance of the concrete, reducing the degree of damage induced by splitting loads, which is a positive improvement in the performance of concrete.

The random distribution of surface cracks in concrete makes it difficult to characterize their distribution features. To address this, fractal dimension has been introduced to quantitatively evaluate the distribution characteristics of splitting cracks on the NMK concrete surface. In this study, the box-counting method was used to determine the fractal dimension (*D*) of the splitting crack distribution in NMK concrete. The value of *D* was calculated using Equations (2)–(4) [38].

(2) $$\frac{N_{i+1}}{N_{i}}={\left(\frac{r_{i}}{r_{i+1}}\right)}^{D},$$

(3) $$N=ar^{D},$$

(4) logN=loga+Dlogr,

where

*N*_i_ = The number of boxes required to cover the cracks;
*r*_i_ = The size of the boxes;
*a* = Coefficient parameter;
*D* = Fractal dimension.

**Figure 10:**
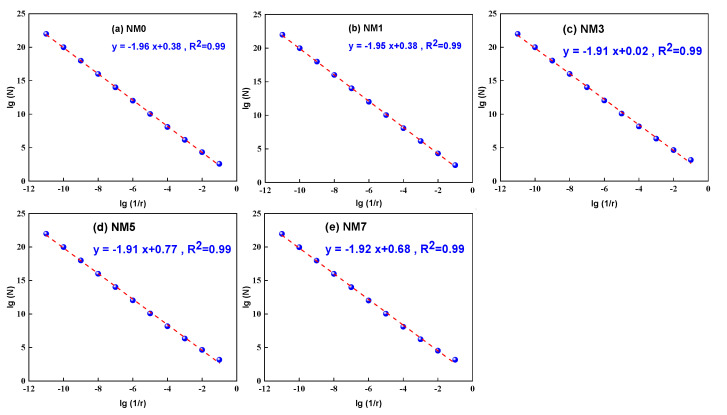
The fractal dimension of NMK concrete.

As demonstrated in Figure 10, a distinct linear relationship is evident between log*N* and log(1/*r*) within all samples, which indicated the presence of pronounced fractal characteristics in the distribution of cracks on the surface of NMK concrete. Following statistical analysis, it was found that the fractal dimension of the surface crack pattern in NMK concrete ranges from 1.9 to 2.0. With an increase in the dosage of NMK, the fractal dimension exhibited a trend of initially decreasing and then increasing. Specifically, the fractal dimensions of surface cracks for NM0, NM1, NM3, NM5, and NM7 are 1.96, 1.95, 1.91, 1.91, and 1.92, respectively. This suggests that the addition of NMK leads to a distribution of surface cracks of concrete that is more consistently and orderly arranged. This phenomenon is attributed to notable enhancement of the interfacial transition zone (ITZ) bond strength [14,17], by filling the voids between mortar and aggregates. This improves the density and overall uniformity of the concrete, leading to cracks tending to propagate along straighter paths, reducing the irregularity and complexity of the crack paths. Additionally, NMK can increase the strength and crack resistance of the cement paste [13,17], further dispersing stress concentrations, reducing the formation of internal cracks in the concrete during splitting tests, and lowering the degree of concrete splitting damage.

**Figure 11:**
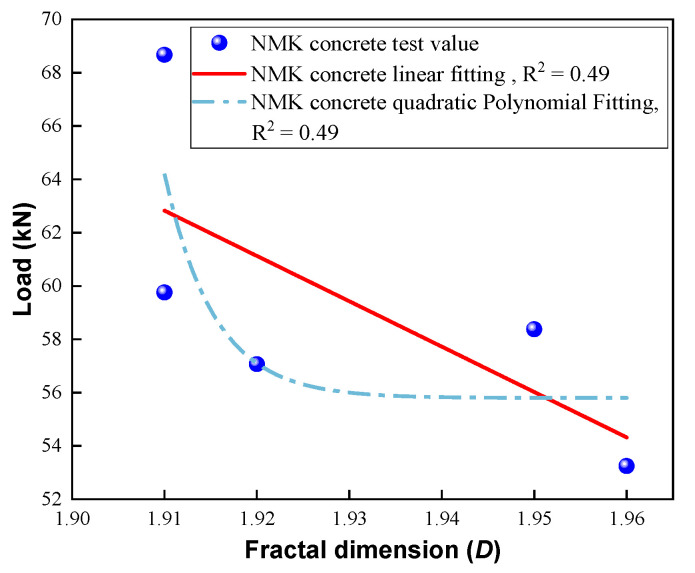
Relationship between fractal dimension of failure cracks and splitting tensile strength.

Furthermore, the relationship between the fractal dimension and the splitting load of concrete is depicted in Figure 11. The fractal dimension and splitting strength in NMK concrete exhibit a pronounced positive correlation, which means that with an increase in the tensile strength of concrete, the fractal dimension of the cracks also tends to increase. This indicates that the distribution characteristics of surface cracks on specimens can reflect the splitting tensile strength of concrete. However, the linear correlation between fractal dimension and splitting tensile strength is relatively weak, with avalue of R2 of 0.48. This observation has been similarly reported by Luan et al. [38]. This is primarily attributed to the heterogeneity within the concrete’s internal components, which results in a random pattern in the propagation of crack paths when subjected to stress [29]. To further investigate the relationship between the fractal dimension of crack distribution and the splitting tensile strength of NMK concrete, a quadratic polynomial fitting analysis was performed. However, a weak quadratic correlation was observed between the fractal dimension of crack distribution and the splitting tensile strength, which may be attributed to the limited number of data points in this study. Future research will explore this relationship more thoroughly based on a larger dataset.

### 3.3. Enhancement Mechanism of Concrete by NMK

#### 3.3.1. XRD

**Figure 12:**
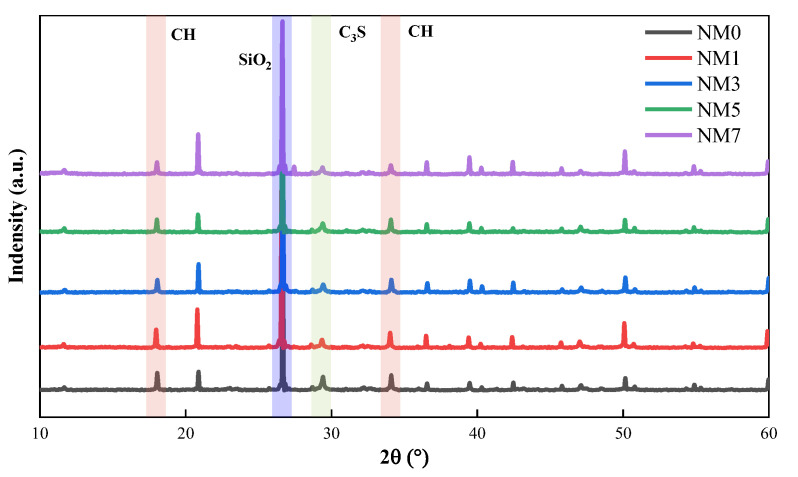
XRD patterns of NMK concrete.

Figure 12 displays the XRD patterns of NMK concrete. Similar diffraction peaks are observed in all specimens, indicate that NMK incorporation does not result in the formation of new hydration products in the concrete. A significant diffraction peak is detected around 25–30°, corresponding to SiO_2_. With the addition of NMK, the intensity of the SiO_2_ diffraction peak increases significantly [39], which relates to the high SiO_2_ content in the NMK composition. The diffraction peak around the 25–30° range is associated with C_2_S and C_3_S in cement. The decreased intensity of the peaks of C_2_S and C_3_S in samples with NMK addition indicates that NMK accelerates the cement hydration reaction, resulting in greater consumption of cement clinker and a higher degree of cement hydration in the concrete. The diffraction peaks around 15–20° and 30–35° are attributed to CH crystals. At 15–20°, the CH diffraction peak intensities for NM1, NM3, NM5, and NM7 are significantly higher than for NM0, with NM1 showing the highest intensity. This observation also confirms that NMK improves the degree of cement hydration. However, as NMK content increases, the CH diffraction peak intensity gradually decreases, primarily due to the pozzolanic reactivity of NMK [14,17], which consumes CH. NM5 exhibits the lowest intensity. However, NM7 exhibits an increase in intensity of CH peak compared to NM5. This may be attributed to the agglomeration of NMK and the dense microstructure formed at an early stage, which reduces the degree of secondary hydration in the concrete [11]. Despite these factors reduce the hydration degree in NM7, its CH diffraction peak intensity still surpasses that of NM0.

#### 3.3.2. FT-IR

**Figure 13:**
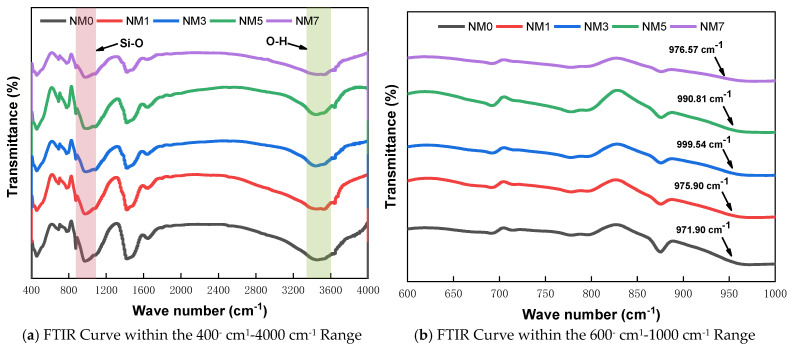
FTIR curves of NMK concrete.

The FT-IR technique is much more sensitive in detecting structural changes in amorphous aluminosilicates, as it is sensitive to short-range structural order [40]. The FT-IR spectrum for NMK concrete is presented in Figure 13. The main band centered at around 950–980 cm^−1^ is attributed to asymmetric stretching vibrations of Si–O–T (T refers to Si or Al) bonds [41]. The second noticeable absorption band appears around 3650 cm^−1^, attributed to the stretching vibrations of the O-H bonds in calcium hydroxide [41]. An absorption peak around 1635 cm^−1^ is also observed, attributed to the H-O-H bending mode of water. Notably, the variation in CH absorption peak intensity is quite similar to that observed in the XRD peaks. With the addition of NMK, the CH absorption peak intensity increases significantly, with NM1, NM3, NM5, and NM7 showing increases of 36.5%, 45.8%, 13.5%, and 57.8% compared to NM0. Additionally, a trend of initial increase followed by decrease is observed in the wavenumber of the C-S-H gel absorption peak as NMK content increased, with the peak shifting towards higher wavenumbers, as illustrated in Figure 13b. This finding suggests that NMK addition leads to the formation of stronger C-S-H gel (C-A-S-H gel), which enhances the mechanical performance of NMK concrete [42].

#### 3.3.3. Microstructure of NMK Concrete

**Figure 14:**
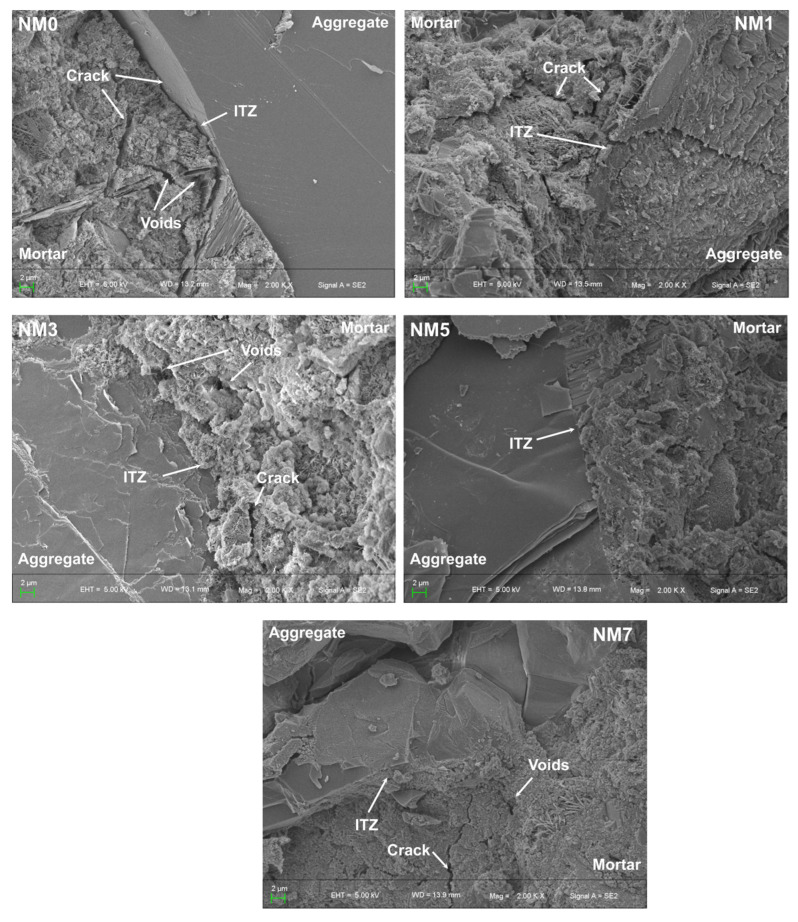
SEM images of NMK concrete.

The primary phases in the microstructure of concrete include the aggregate, mortar, and the interfacial transition zone (ITZ) between the aggregate and mortar. The ITZ is the most crucial phase, profoundly affecting the properties of concrete [43]. The mortar matrix and ITZ phases were examined using SEM images to investigate the mechanism by which NMK affects the tensile strength of concrete. Figure 14 illustrates the ITZ microstructure in NMK concrete. A clear ITZ between mortar and aggregate are observed. The NM0 presents a loose, porous microstructure with inadequate contact between aggregate and mortar, alongside several pores and cracks near the ITZ. This microstructure is a typical observation in concrete [43]. Compared to NM0, the concrete with NMK shows a denser ITZ structure and fewer cracks. This improvement in microstructure is the primary reason for the enhanced tensile strength of NMK concrete. In addition, a disordered arrangement of hydration products is observed in NM0, especially around the ITZ. This is mainly due to the larger pores near the ITZ, providing ample space for random growth of hydration products like CH crystals and AFt. With the incorporation of NMK, the hydration products gradually become more ordered, forming a network structure. This is mainly due to the nucleation effect of NMK, which offers attachment sites for the hydration products, improving their arrangement [43]. Moreover, the smaller NMK particles fill the ITZ pores, restricting the space for random growth of hydration products. This optimized arrangement improves the crack resistance of NMK concrete. However, at higher NMK content (7 wt.% NMK), although NM7 exhibits a dense microstructure, distinct cracks are also observed near the ITZ. This may be due to the agglomeration of NMK, which weakens the benefit effect of NMK on concrete and contributes to stress concentration around the agglomerates during cracking, leading to lower splitting tensile strength in NM7. This finding aligns with the conclusion in Section 3.1.

**Figure 15:**
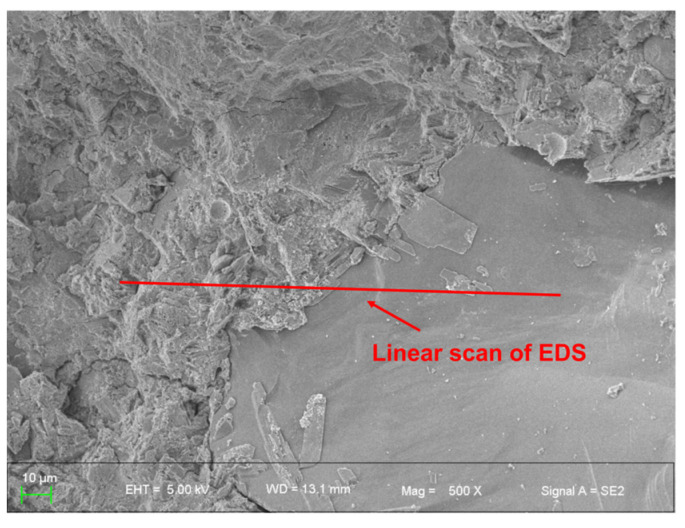
Schematic diagram of EDS line scan position.

The analysis of the EDS maps of the concretes emphasized that the semiquantitative analysis of the proportions of the chemical elements is valid for studying the microstructure, especially the ITZ [44]. In the case of the Ca/Si atomic mass ratios, the instability of the values in the region close to the aggregate, ITZ, was more evident, and its thickness could be measured. Figure 15 illustrates the schematic representation of the EDS line scan for NM0.

**Figure 16:**
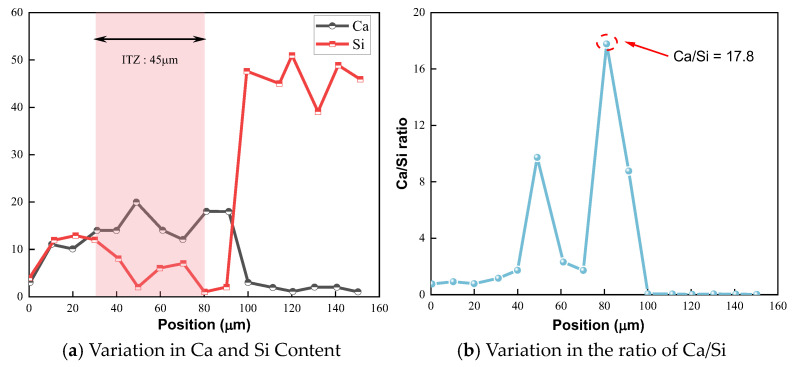
Distribution Patterns of Ca and Si in NM0.

As illustrated in Figure 16, it can be observed from Figure 16a that the Ca/Si ratio in the section of mortar generally remains within the range of 0 to 2. As the EDS scanning position shifts towards the ITZ, the Ca content increases significantly, while the Si content shows an opposite trend. This can mainly be attributed to the substantial growth of CH crystals in the ITZ region [44], resulting in an elevated Ca content [43,45]. As the scanning position continues towards the aggregate, the Ca content rapidly declines, while Si content swiftly rises, indicating that this area pertains to the aggregate part, where the high Si content stems from the SiO_2_ present in the aggregate. The ITZ thickness of NM0 is around 45 μm, which aligns with previous research outcomes [43]. Additionally, NM0 exhibits a maximum Ca/Si ratio of 17.8, indicating fewer C-S-H gels and an increased presence of CH crystals [43,45], which results in reduced splitting tensile strength of NM0. Furthermore, the morphology and size of CH crystals favor crack propagation, thereby further weakening crack resistance of NM0.

**Figure 17:**
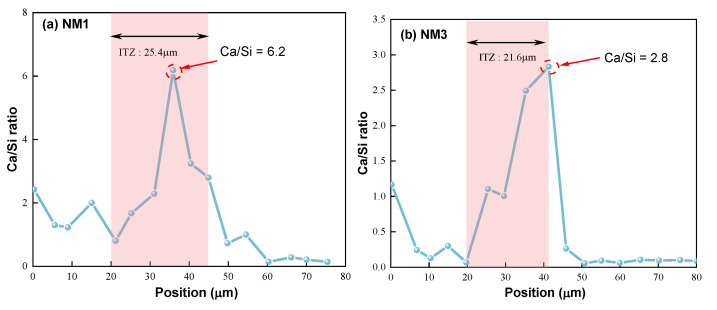

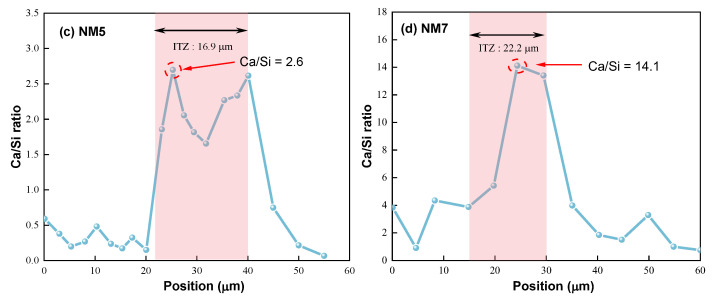
Distribution Characteristics of Ca and Si in NMK Concrete.

The distribution of the Ca/Si ratio in NMK concrete is shown in Figure 17. A similar Ca/Si ratio variation curve to that of NM0 was observed in all NMK concretes. The results indicate that the addition of NMK notably decreases the thickness of the ITZ and the maximum Ca/Si ratio in this region. Specifically, the ITZ thicknesses for NM1, NM3, NM5, and NM7 are 25.4 μm, 21.6 μm, 11.3 μm, and 22.2 μm, respectively, representing reductions of 43.6%, 52%, 62.4%, and 50.7% compared to NM0. This reduction is mainly attributed to the pore refinement ability of NMK, which results in a denser ITZ microstructure, thereby enhancing the tensile properties of the concrete. A lower value of Ca/Si ratio typically indicates that the CSH gel has higher strength. Compared to NM0, the maximum Ca/Si ratios for NM1, NM3, NM5, and NM7 decreased by 65.2%, 84.3%, 85.4%, and 20.8%, respectively. These lower Ca/Si ratios indicate better development of C-S-H gel in all NMK concretes, which could be the reason for their excellent tensile properties. However, with higher NMK content (>5%), the Ca/Si ratio of NM7 increased by about four times compared to NM5. This phenomenon further demonstrates that excessive NMK reduces the extent of secondary hydration, decreases the formation of high-strength CSH, and consequently results in reduced tensile performance of the concrete.

#### 3.3.4. TG

**Figure 18:**
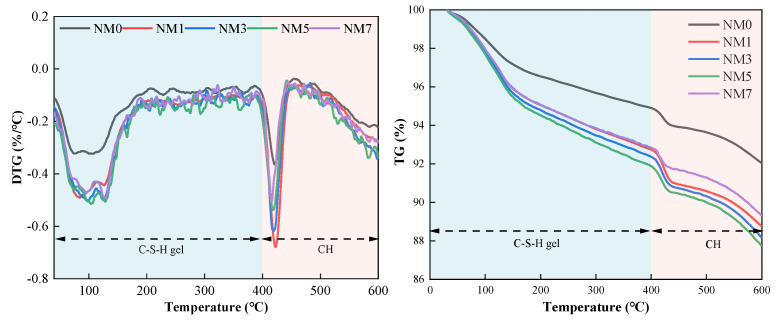
TG-DTG curves of concrete with NMK content.

The results of TG of NMK concrete, are depicted in Figure 18. A consistent trend of mass loss was observed in all samples. These curves display two major mass loss peaks for samples. The peak of mass loss between 0 °C and 200 °C is attributed to the decomposition of C-S-H gel [39], while the peak between 400 °C and 600 °C corresponds to the decomposition of calcium hydroxide (CH) [39]. NM0 exhibits a mass loss of approximately 3.5% within the (0–200) °C range. As the NMK content increases, the mass loss of C-S-H gel progressively increases, indicating an enhancement in C-S-H gel formation, likely attributable to the pozzolanic effect of NMK [14,17]. Notably, NM5 displays the highest mass loss (5.5%) of CSH gel, representing a 57.1% increase over NM0. Within the (400–600) °C range, the mass losses of CH for NM0, NM1, NM3, NM5, and NM7 are 1.3%, 2.2%, 2.0%, 1.9%, and 1.6%, respectively. The incorporation of NMK also enhances mass loss of CH, which can be attributed to nucleation effect [12] of NMK in promoting cement hydration. However, a decreasing trend is observed with further increases in NMK content. This is consistent with the results obtained from XRD. This phenomenon further confirms the pozzolanic effect of NMK, as the consumption of CH crystals and the generation of additional hydration products [11]. Additionally, NM7 exhibits lower mass losses of CH and C-S-H compared to NM5. This suggests that excessive NMK may reduce the degree of cement hydration.

## 4. Conclusions

This study conducted splitting tensile tests on NMK concrete and obtained surface strain and deformation data using Digital Image Correlation (DIC) method. The primary focus was on analyzing the failure behavior of NMK concrete. Additionally, the mechanisms by which NMK influences the splitting tensile performance of concrete were explored through X-ray Diffraction (XRD), Fourier Transform Infrared Spectroscopy (FT-IR), and Thermogravimetric Analysis (TG). The impact of NMK on the interfacial transition zone between aggregate and mortar was examined using Scanning Electron Microscopy/Energy Dispersive Spectroscopy (SEM/EDS). The following conclusions were drawn:

1. NMK enhances the splitting tensile strength of concrete. Concrete with 5% NMK addition exhibited a 17.4% increase in splitting tensile strength compared to ordinary concrete.
2. NMK improves the crack resistance of concrete. Concrete with 5% NMK addition showed reductions in crack length and width by 4.5% and 35.3%, respectively, compared to ordinary concrete. Furthermore, the crack propagation rate decreased by 29.6%.
3. NMK reduces the thickness of the aggregate-mortar interfacial transition zone (ITZ) and the Maximum Ca/Si Ratio in the ITZ. Concrete with 5% NMK addition exhibited a 62.4% reduction in ITZ thickness and an 85.4% reduction in the maximum Ca/Si ratio compared to ordinary concrete.

## Figures and Tables

**Table 1:** Physical index of NMK.

Average Flake Thickness (nm)	Specific Surface (m^2^/g)	Density (g/cm^3^)	Activity Index (%)	Whiteness (%)
30	4.09	2.47	120	80

**Table 2:** Chemical composition of cement and NMK (wt.%).

Materials	SiO_2_	Al_2_O_3_	Fe_2_O_3_	CaO	MgO	SO_3_	K_2_O	Na_2_O	TiO_2_	LOI
Cement	21.91	6.27	3.78	59.30	1.64	2.41	-	-		4.69
NMK	49.40	42.88	0.51	0.27	2.66	0.14	0.23	1.52	0.04	2.17

**Table 3:** Mixture proportions of NMK concrete.

Mixture ID	Amount of Concrete Material (kg/m^3^)				
	Cement	Sand	Gravel	Water	NMK
NM0	450	541	1200	200	0
NM1	445.5	541	1200	200	4.5
NM3	436.5	541	1200	200	13.5
NM5	427.5	541	1200	200	22.5
NM7	418.5	541	1200	200	31.5

**Table 4:** The propagation velocity of main crack of NMK concrete with different NMK content.

Specimens ID	Length of Main Crack (mm)	The Frames from Initiation to Penetration of Main Crack (fps)	Relative Propagation Velocity of Main Crack (mm/fps)
NM0	106.20	44	2.41
NM1	103.26	47	2.20
NM3	103.32	51	2.01
NM5	101.44	52	1.93
NM7	100.64	42	2.40

## Data Availability

The original contributions presented in the study are included in the article, further inquiries can be directed to the main authors.
